# An Efficient Resource Management Optimization Scheme for Internet of Vehicles in Edge Computing Environment

**DOI:** 10.1155/2022/3207456

**Published:** 2022-05-28

**Authors:** Anqing Zhu, Youyun Wen

**Affiliations:** Management School, South China Business College Guangdong University of Foreign Studies, Guangzhou, Guangdong 510000, China

## Abstract

The contradiction between limited network resources and a large number of user demands in vehicle environment will cause a lot of system delay and energy consumption. To solve the problem, this paper proposes an efficient resource management optimization scheme for Internet of Vehicles in edge computing environment. Firstly, we give a detailed formulation description of communication and computing cost incurred in the resource optimization process. Then, the optimization objective of this paper is clarified by considering the constraints of computing resources, and system delay and energy consumption are considered comprehensively. Secondly, considering dynamic, random, and time-varying characteristics of vehicle network, the optimal resource management scheme of Internet of Vehicles is given by using distributed reinforcement learning algorithm to optimize total system overhead to the greatest extent. Finally, experiments show that when bandwidth = 40 MHz, the total system cost of the proposed algorithm is only 3.502, while that of comparison algorithms is 4.732 and 4.251, respectively. It is proved that the proposed method can effectively reduce the total system overhead.

## 1. Introduction

In recent years, the automotive industry has developed rapidly, and intelligence and networking have become an important trend in the future development of automotive industry [1]. On the one hand, these technologies enable communication and information exchange between Vehicle to Vehicle (V2V) and Vehicle to Infrastructure (V2I), helping to build safe, collaborative, and intelligent transportation systems. On the other hand, this in turn generates a large amount of data, and at the same time, the demand for computing, communication, and content increases significantly [2–4]. With the development of Internet of Vehicles (IoV) and intelligent connected vehicles, in-vehicle infotainment applications such as road safety, intelligent navigation, autonomous driving, and in-vehicle entertainment continue to emerge. This promotes the development of intelligent transportation and brings a great improvement to driving experience [5–7]. Due to the particularity of the physical location of vehicles and cloud servers, the backhaul link capacity is limited. Such a high content demand of the Internet of Things will bring a huge burden to the core network [9]. At the same time, they also pose a major challenge to support massive content delivery and meet the low-latency requirements of IoT [10–12].

The introduction of mobile edge computing (MEC) technology makes up for the network instability and delay limitations of cloud computing in IoT scenario and is more suitable for low-latency, high-reliability task computing on IoT requirements [13–18]. The cloud server located in core network is far away from vehicles, and vehicles need to rely on a large base station for multi-hop transmission to offload tasks to the cloud server for processing. However, it is prone to network fluctuations and transmission interruptions and is unreliable for in-vehicle applications, especially safe driving applications [19, 20]. Therefore, using distributed MEC services to replace traditional cloud computing services can effectively solve the resource management optimization problem in IoT [21].

The main factors that affect the decision of computing offloading are the execution delay and energy consumption of task. Thus, optimization goals usually include solutions such as reducing delay, reducing energy consumption, and weighting between delay and energy. Reinforcement learning can capture the hidden dynamics of environment well, so it is often used to optimize resource allocation algorithms. Liu et al. [22] proposed a resource allocation strategy based on deep reinforcement learning (DRL). Zhan et al. [23] designed a strategy optimization method based on DRL by using game theory. Huang et al. [24] studied the wireless charging MEC network and proposed an online decision-making method based on DRL. Hui et al. [25] proposed a content dissemination framework based on edge computing. Combining the selfishness and transmission ability of vehicles, the authors designed a two-level relay selection algorithm to reasonably select relay vehicles to meet different transmission needs. Su et al. [26] used the vehicles parked around the street and the vehicles driving along the road to communicate the vehicle social community through V2V and used the content cached in the parked vehicles to reduce the delay of content download. Zhao et al. [27] proposed a caching strategy in V2V scenario with information as the center and designed a dynamic probabilistic caching scheme. Zhang et al. [28] proposed a MEC scenario computing resource allocation scheme based on DRL network, which avoids falling into the disaster of dimensionality. Zhang et al. [29] proposed a joint optimization scheme of IoT content caching and resource allocation based on MEC in high-speed free-flow scenario, which reduced data acquisition latency. Li [29] proposed a resource allocation strategy for computing unloading in vehicle Internet based on DRL. However, in the case of limited network resources and a large number of user demands, the above research has the problems of excessive resource delay consumption and large energy consumption. Therefore, the optimization of IoT resource management in the MEC system scenario is a challenging problem.

Based on the above analysis, in view of delay and energy consumption caused by the contradiction between limited network resources and a large number of user needs in vehicle environment, this paper proposes an efficient resource management optimization scheme for IoT in edge computing environment. This method takes minimizing the weighted sum of system delay and energy consumption as the optimization goal and constructs a communication model and task offloading optimization model in IoT edge computing scenario. Moreover, a solution algorithm based on distributed reinforcement learning is used to optimize the total system overhead.

## 2. System Model and Problem Modeling

### 2.1. System Model

**Figure 1:**
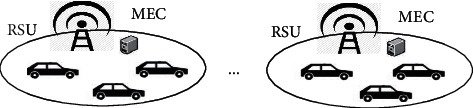
IoT system model.

The system model is shown in Figure 1. *J* road side units (RSUs) are evenly distributed on the road, and all have MEC services configured, denoting MEC server as mec_*j*_, *j* ∈ {1,2,…, *J*}. Each of *C* randomly distributed vehicles performs multiple computing tasks. Suppose the sum of computing tasks of all vehicles is *N*, and the computing tasks are denoted by *L*. *b* represents the size of input data, *w* represents the task computation amount, and *t*^max^ represents the task deadline. If the task processing exceeds the time limit, it means that the task processing fails, and *R*_*L*_ represents MEC cell carried by vehicle-mounted terminal to which the task belongs. *ω* represents the importance of computing task to distinguish the task is a secure computing task and a common computing task. Therefore, the computational task can be denoted as *L*={*b*, *w*, *ω*, *t*^max^, *R*_*L*_}. Let *i* represent the number of onboard terminals offloading the computing task to MEC server, and *x*=0 indicates that the task is executed locally. The offloading strategies of N computing tasks constitute the offloading strategy vector set *X*={*x*_1_, *x*_2_,…, *x*_*N*_}.

### 2.2. Communication Model

When task *i* chooses to perform computing offloading, a corresponding offloading decision needs to be made to decide which MEC server to offload to and which channel to select to upload data. When the offloading decision vector **d** of all users is given, data transmission rate *R*_*n*_^*m*^(**d**) on the *n* channel between user *u*_*i*_ of offloading decision *d*_*n*_^0^ > 0 and the *j* RSU can be obtained. The maximum information transfer rate *V* is

(1) $$\begin{array}{c} V=W\;\log_{2}\left(1+\text{SNR}\right), \end{array}$$

where *W* is the channel bandwidth and SNR is the ratio of average power of signal transmitted in the channel to noise power in the channel, that is, the signal-to-noise ratio. Its calculation is

(2) $$\begin{array}{c} \text{SNR}=\frac{p_{i}g_{i}^{h}}{\gamma^{2}+\sum_{i\in V}p_{i}g_{i}^{h}}, \end{array}$$

where *p*_*i*_ represents the transmission power of users, that is, the transmit power of user equipment; *g*_*i*_^*h*^ represents the channel gain of communication channel selected by users; and *γ*^2^ represents the white Gaussian noise power.

The data transfer rate *V*_*i*,*j*_^*h*^(*d*) is

(3) $$\begin{array}{c} V_{i,j}^{h}\left(d\right)=B\;\log_{2}\left(1+\frac{p_{i}g_{i}^{h}}{\gamma^{2}+\sum_{i\in V}p_{i}g_{i}^{h}}\right). \end{array}$$

### 2.3. Computing Offloading Model

#### 2.3.1. Local Computing

Assuming that task *i* is only calculated locally, only the calculation delay is considered. *e*^loc^ represents the computing capability of vehicle terminals. The processing delay of local tasks and the energy required for local computing are expressed as

(4) $$\begin{array}{c} t_{i}=t_{\text{loc}}=\frac{w_{i}}{e^{\text{loc}}},\\ E_{i}=t_{i}P_{\text{loc}}. \end{array}$$

When MEC server resources are insufficient, the system unloads the task to other servers.

#### 2.3.2. Local Server Computing

When the vehicle communicates directly with the local server, the vehicle will unload the computing task to the server in the cell. After the server completes the execution, the result will be returned to the vehicle immediately. The total task delay includes upload delay, server calculation delay, and return delay. Let *t*_*i*,*j*_^mec^ denote the task execution delay, *e*_*i*,*j*_^mec^ denote the computing resources, and *v*_*i*,*j*_ denote the wireless transmission rate:

(5) $$\begin{array}{c} t_{i,j}^{\text{mec}}=\frac{w_{i}}{e_{ij}^{\text{mec}}},\\ t_{i,j}^{\text{trans}}=\frac{b_{i}}{v_{ij}}. \end{array}$$

Since the return rate is much higher than the upload rate, the return delay of the calculation result can be ignored. The total time delay of unloading calculation is

(6) $$\begin{array}{c} t_{i,j}=t_{i,j}^{\text{trans}}+t_{i,j}^{\text{mec}}. \end{array}$$

The energy consumption for unloading calculation is

(7) $$\begin{array}{c} E_{i,j}=P_{\text{loc}}\left(t_{i,j}^{\text{trans}}+t_{i,j}^{\text{mec}}\right), \end{array}$$

where *P*_loc_ represents the energy consumption per unit cycle of local CPU.

#### 2.3.3. Other Server Computing

When the MEC server in the cell where the vehicle is located is overloaded, the computing task is unloaded to other cell servers. Communication between MEC servers is generally performed through wired communication links such as optical fibers. Assuming that the average task transmission delay on the wired link is *t*_*w*_ and *c* represents the number of wired link hops between computing tasks offloaded to other servers, the task processing delay at this time is expressed as

(8) $$\begin{array}{c} t_{i,j}^{o}=2ct_{w}+t_{i,j}^{\text{trans}}+\frac{w_{i}}{e_{i,j}^{\text{mec}}}. \end{array}$$

Then, the energy consumption of other servers' offloading computing is

(9) $$\begin{array}{c} E_{i,j}^{o}=P_{\text{loc}}\left(2ct_{w}+t_{i,j}^{\text{trans}}+\frac{w_{i}}{e_{i,j}^{\text{mec}}}\right). \end{array}$$

### 2.4. Problem Modeling

*τ* is the calculated weight, and the weighted sum of the total delay is

(10) $$\begin{array}{c} t_{\text{all}}=\sum_{i=1}^{N}\left(\tau t_{i}+\left(1-\tau \right)\sum_{j=1}^{J}t_{ij}\right). \end{array}$$

The total energy consumption is

(11) $$\begin{array}{c} c_{\text{all}}=\sum_{i=1}^{N}\left(\tau C_{i}+\left(1-\tau \right)\sum_{j=1}^{J}t_{ij}\right). \end{array}$$

Considering delay and energy consumption, the total cost of local calculation is

(12) $$\begin{array}{c} C_{all}=\gamma t_{\text{all}}+\left(1-\gamma \right)c_{\text{all}}, \end{array}$$

where *γ* is the weight.

The optimization problem can be formulated as

(13) $$\begin{array}{c} \min\left(C_{\text{all}}\right). \end{array}$$

In order to ensure that the task is completed on time, the calculation task is required to complete the task before the vehicle leaves the MEC unit, and the following conditions shall be met:

(14) $$\begin{array}{c} t_{i}^{\text{stay}}=\frac{s_{i}}{v_{i}},\\ t+\frac{w_{i}}{e_{i,j}^{\text{mec}}}\leq \min\left[t^{\max},t_{i}^{\text{stay}}\right]. \end{array}$$

Computing tasks unloaded to other servers should meet the following conditions:

(15) $$\begin{array}{c} 2ct_{w}+t_{i,j}^{\text{trans}}+\frac{w_{i}}{e_{i,j}^{\text{mec}}}\leq \min\left[t^{\max},t_{i}^{\text{stay}}\right]. \end{array}$$

The computing resources required to complete computing tasks are

(16) $$\begin{array}{c} e_{i,j}^{\text{mec}}\geq \max\left\{\frac{w_{i}}{\min\left[t^{\max},t_{i}^{\text{stay}}\right]-t_{i,j}^{\text{trans}}},\frac{w_{i}}{\min\left[t^{\max},t_{i}^{\text{stay}}\right]-2ct_{w}-t_{i,j}^{\text{trans}}}\right\}. \end{array}$$

The total computing resources required for computing tasks are

(17) $$\begin{array}{c} e_{j}=\sum_{i=1}^{N}\sum_{x_{i}=j}e_{i,j}^{\text{mec}}. \end{array}$$

The constraints are as follows:

(18) $$\begin{array}{c} C1:x_{i}\in \left\{0,1,2,\ldots ,j\right\},\forall i\in N,\\ C2:y_{i}\in \left\{0,1\right\},\forall i\in N,\\ C3:e_{j}<E_{j},j\in \left\{1,2,\ldots ,J\right\}. \end{array}$$

C1 indicates that a computing task can only be offloaded to one edge server and cannot be offloaded to two or more at the same time. C2 means that the computing task adopts binary offloading, which can choose not to offload or offload entire task at the same time, that is, the task is indivisible. C3 indicates that computing resources required by computing tasks offloaded to edge server cannot exceed the total resources of edge servers.

## 3. Solutions Based on Reinforcement Learning

### 3.1. Problem Solving Based on Distributed Reinforcement Learning

In view of dynamic, random, and time-varying nature of in-vehicle networks, artificial intelligence algorithms are more suitable for resource management and task scheduling than traditional mathematical methods. In comparison, Q-learning needs to maintain Q-table and is not suitable for networks with many states. Deep deterministic policy gradient algorithms need to use an experience replay mechanism to eliminate the correlation between training data. For experience playback mechanism, the agent consumes more resources for each interaction with the environment. The off-policy learning method adopted can only be updated based on the data generated by the old policy. Therefore, consider using the actor-critic algorithm to reduce the overhead required for algorithm execution, while providing optimal offloading decisions and resource management based on real-time network environment. Modeling the environment of system with an actor-critic algorithm requires determining its state space, action space, and reward function.

The state space, *S*, consists of computing resources and cache resources of in-vehicle network, *S*={*F*_1_, *F*_2_,…, *F*_*M*_, *S*_1_, *S*_2_,…, *S*_*M*_}, where *F*_*i*_ and *S*_*i*_ represent the computing capacity and storage capacity of road side unit *i*, respectively.

The action space consists of offloading decisions of vehicles, caches of road side units, and computing resource management, *A*=(*x*_*i*_, *w*_*i*_, *f*_*i*_), where *x*_*i*_={*x*_*i*0_, *x*_*i*1_,…, *x*_*iM*_}, *w*_*i*_={*w*_*i*1_, *w*_*i*2_ …, *w*_*iM*_}, and *f*_*i*_={*f*_*i*1_, *f*_*i*2_ …, *f*_*iM*_} represent the set of vehicle *i* offloading decision, road side unit storage, and computing resource management, respectively.

*Reward Function*. The goal of reinforcement learning training is to maximize long-term cumulative reward. According to the objective function of this paper, the reward function is designed as

(19) $$\begin{array}{c} r_{i,t}=1-\frac{C_{i,j}}{\max\left\{C_{i,j}\right\}}. \end{array}$$

The public neural network in actor-critic algorithm consists of multiple threads, and each thread has the same 2 modules as public neural network: the policy (actor) network and the critic (critic) network. The actor network is used to optimize the policy *π*(*a*_*t*_|*s*_*t*_; *δ*) with parameters *δ*; the critic network tries to estimate the value function *V*(*s*_*t*_; *δ*) with parameters *δ*_*v*_. At time *t*, the actor network performs action *a*_*t*_ based on current state *s*_*t*_, gets a reward *r*_*t*_, and enters the next state *s*_*t*+1_.

Use the advantage function *A*(*a*_*t*_, *s*_*t*_) to represent the difference between the action value function *Q*(*a*_*t*_, *s*_*t*_) and state value function *V*(*s*_*t*_):

(20) $$\begin{array}{c} A\left(a_{t},s_{t}\right)=Q\left(a_{t},s_{t}\right)-V\left(s_{t}\right). \end{array}$$

To speed up convergence, approximate *Q*(*a*_*t*_, *s*_*t*_) with *k* step sampling:

(21) $$\begin{array}{c} Q\left(\left(a_{t},s_{t}\right)\right.\approx \sum_{i=0}^{k-1}\gamma^{i}r_{t+i}+\gamma^{k}V\left(s_{t+k};\delta_{v}\right), \end{array}$$

where *γ* is the discount coefficient, *r*_*t*+*i*_ represents the instant reward, and *V*(*s*_*t*_) is obtained through critic network.

Taking the parameter *δ* as a variable, differentiate the policy loss function to obtain

(22) $$\begin{array}{c} \nabla_{\delta }f_{\pi }\left(\delta \right)=\nabla_{\delta }\log\;\pi \left(a_{t}||s_{t};\delta \right)A\left(a_{t},s_{t}\right)+\beta \nabla_{\delta }H\left(\pi \left(s_{t};\delta \right)\right), \end{array}$$

where *H* is the entropy of policy and *β* is the coefficient.

For the value loss function, there are

(23) $$\begin{array}{c} f_{v}\left(\delta_{v}\right)={\left(R_{t}-V\left(s_{t};\delta \right)\right)}^{2}. \end{array}$$

Based on RMSProp algorithm, the gradient estimate can be expressed as

(24) $$\begin{array}{c} g=ag+\left(1-a\right)\Delta \delta^{2}, \end{array}$$

where *a* represents momentum and Δ*δ* represents the cumulative gradient of loss function.

The update parameters of RMSProp algorithm are

(25) $$\begin{array}{c} \delta \leftarrow \delta -\eta \frac{\Delta \delta }{\sqrt{g+\varepsilon }}. \end{array}$$

### 3.2. Algorithm Flow

The proposed offloading strategy flow based on distributed reinforcement learning is shown in Algorithm 1.

## 4. Example Verification and Result Discussion

### 4.1. Simulation Settings

This section uses Python to simulate and verify resource management optimization scheme for IoT and evaluate the pros and cons of different algorithms by comparing the impact of each algorithm on the total system overhead with the number of vehicles, the number of tasks, and the bandwidth. The simulation parameters are set as shown in Table 1. Due to the existence of small-scale fast fading and the mobility of mobile devices in established model, the results of each run are random. Therefore, the mathematical method of statistical averaging is used to obtain average value as the final result. The computer configuration information used for the simulation is Windows Server 2019, Intel(R) Xeon(R) 2.6 GHz processor, and 16 GB RAM.

### 4.2. Convergence Performance Analysis

**Figure 2:**
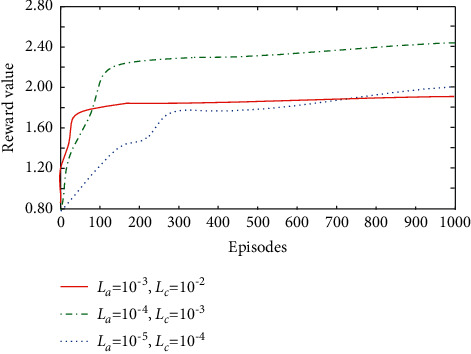
Performance comparison under different learning rates.

Figure 2 describes the convergence of algorithm in this paper under different learning rate scenarios. It can be found from the figure that when the learning rates of actor and critical networks are *L*_*a*_  = 1×10−3 and *L*_*b*_  = 1×10−2, respectively, although the learning speed of algorithm is very fast, it will degrade the final convergence performance of system. When the learning rate is too small (*L*_*a*_  = 1×10−3, *L*_*b*_  = 1×10−2), the learning speed will drop sharply. Therefore, the learning rate is set to *L*_*a*_  = 1×10−4, *L*_*b*_  = 1×10−3 in the follow-up experiment.

### 4.3. Comparison of Accumulated Average Rewards under Different Schemes

**Figure 3:**
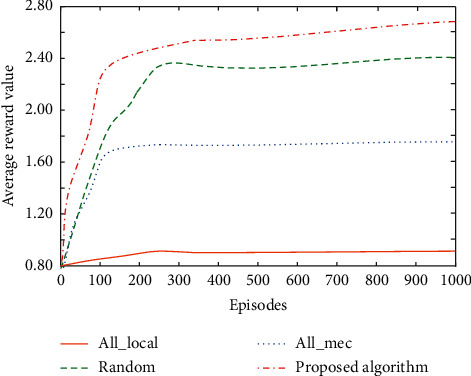
Comparison of average rewards under different schemes.

Compare the average reward value of the proposed scheme with the following schemes: (1) all-local strategy; (2) random strategy; (3) all-MEC policy. During DDPG training, there will be violent shocks. Thus, this section observes the convergence of neural network by calculating the cumulative average value of system reward.  Figure 3 shows the comparison of cumulative average rewards for different caching schemes. With the increase of training times, it can be seen that all-MEC and random schemes can gradually converge to a stable cumulative average. All-local strategy behavior is not encouraged, so the reward value is the lowest. Because the proposed algorithm needs to consider the road conditions of adjacent areas, increases the dimension of system state, and improves the complexity, it has poor performance at the beginning of training and obtains the highest average reward value after convergence. Therefore, the proposed resource optimization management scheme for IoT can make full use of communication resources and effectively improve the effectiveness of the system.

### 4.4. Performance Comparison under Different Algorithms

**Figure 4:**
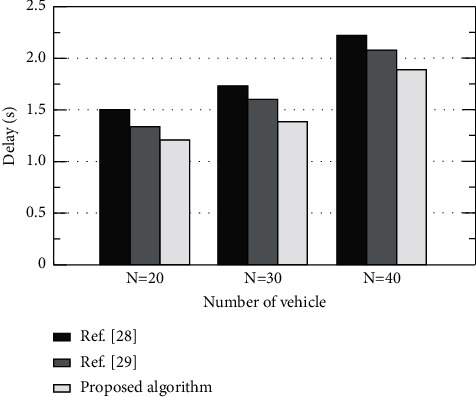
Influence of vehicle number on delay of different algorithms.

In order to prove the advantages of the proposed algorithm, the algorithms in [28–29] are compared with the proposed algorithm under the same experimental conditions.  Figure 4 shows the impact of the number of vehicles on the delay. It can be found that the delay of system task processing increases with the increase of the number of vehicles. This is mainly due to the increase of processing tasks and the limitation of computing resources. Among all algorithms, The reference [29] algorithm has the largest delay. Compared with the reference [29] algorithm and the proposed algorithm, the vehicle will undertake more tasks. Due to the limitation of the vehicle's own computing resources, processing tasks alone will cause greater delay. Due to the limitation of vehicle computing resources, processing tasks alone will cause large time delay. The proposed algorithm considers the cooperation of terminal, edge, and cloud, improves the utilization efficiency of resources, and minimizes the system delay.

**Figure 5:**
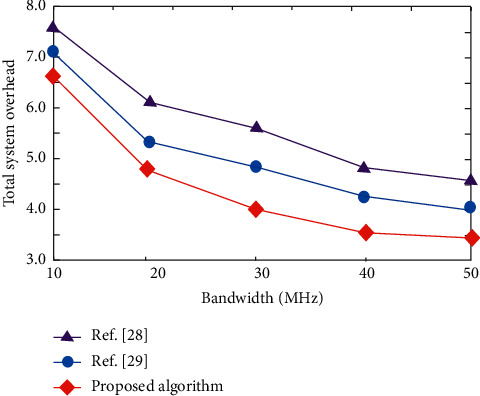
Variation of total system overhead with bandwidth under different algorithms.

The change of total system overhead with bandwidth under different algorithms is shown in Figure 5. Figure 5 shows that with the increase of bandwidth, the total system overhead of three algorithms shows a downward trend, but the total system overhead of the proposed algorithm is always lower than that of other two algorithms. When bandwidth = 40 MHz, the total system overhead of the algorithm in [28] is 4.732, and the total system overhead of the algorithm in [28] algorithm is 4.251, while the total system overhead of the proposed algorithm is only 3.502. Further analysis shows that when the cloud computing ability of comparison algorithm is relatively weak, most of computing tasks will be completed at the edge node, which cannot make good use of the cloud edge system. Therefore, it produces high total system overhead. Compared with the other two algorithms, the proposed algorithm can achieve the lowest total system overhead because the proposed algorithm considers the dynamic, random, and time-varying characteristics of vehicle network to optimize system performance to the greatest extent.

**Figure 6:**
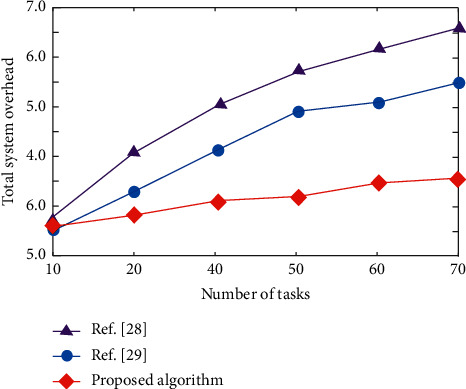
Changes of total system overhead with the number of tasks under different algorithms.

The change of total system overhead with the number of tasks is shown in Figure 6. With the increase of the number of tasks, the total cost of the three algorithms shows an upward trend. However, the total system overhead of this algorithm is less than that of the algorithms in [28, 29]. This is because the algorithm can collect the state and action information of the whole system and make better decisions according to the global information, so the total cost of the system is low. The comparison algorithm does not fully analyze the state and action information of the system, and the multi-vehicle game increases the energy consumption, resulting in the increase of the total cost of the system.

## 5. Conclusion

Aiming at the problem of delay and energy consumption caused by the contradiction between limited network resources and a large number of user needs in vehicle environment, this paper proposes an efficient resource management optimization scheme for IoT in edge computing environment. The proposed algorithm builds a communication model and task offloading optimization model in IoT edge computing scenario and solves them based on distributed reinforcement learning to maximize the system performance.

In the future, we will study the content acquisition decision combined with micro-traffic data and the prediction of vehicle mobility to further improve algorithm performance. Besides, a dynamic situation will be considered, that is, devices may leave the current edge server during computing offloading. In this case, it is necessary to set up a more effective mobile model for devices. In addition, the current blockchain technology provides a powerful solution for the unloading of secure computing tasks in the IoV. In view of the contradiction between the high real time of IoV applications and the low real time of blockchain, we can take advantage of the differences of participants in IoV in terms of security level, computing power, and communication ability to design a hierarchical blockchain structure matching the cloud IoV structure to solve it. It is of great significance to use the blockchain technology to build a secure Internet of Vehicles computing task unloading platform.

## Figures and Tables

**Algorithm 1:**
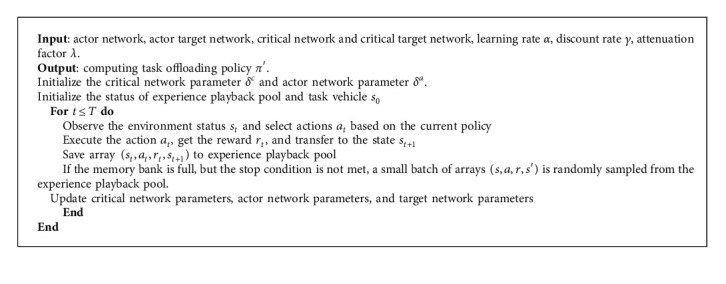
Resource management algorithm based on distributed reinforcement learning.

**Table 1:** System parameters.

Parameter	Value
Number of concurrent tasks of a single vehicle	2∼7
Number of vehicles	5∼15
Calculation capability of onboard terminal	5 MHz
Vehicle transmission power	1.5 W
Vehicle speed	30–80 KM/h
Gaussian white noise power	-80 dB
MEC computing power	[2 × 10^8^, 9 × 10^8^]Hz
System bandwidth	10∼50 MHz

## Data Availability

The data used to support the findings of this study are included within the article.
